# Four principles to establish a universal virus taxonomy

**DOI:** 10.1371/journal.pbio.3001922

**Published:** 2023-02-13

**Authors:** Peter Simmonds, Evelien M. Adriaenssens, F. Murilo Zerbini, Nicola G. A. Abrescia, Pakorn Aiewsakun, Poliane Alfenas-Zerbini, Yiming Bao, Jakub Barylski, Christian Drosten, Siobain Duffy, W. Paul Duprex, Bas E. Dutilh, Santiago F. Elena, Maria Laura García, Sandra Junglen, Aris Katzourakis, Eugene V. Koonin, Mart Krupovic, Jens H. Kuhn, Amy J. Lambert, Elliot J. Lefkowitz, Małgorzata Łobocka, Cédric Lood, Jennifer Mahony, Jan P. Meier-Kolthoff, Arcady R. Mushegian, Hanna M. Oksanen, Minna M. Poranen, Alejandro Reyes-Muñoz, David L. Robertson, Simon Roux, Luisa Rubino, Sead Sabanadzovic, Stuart Siddell, Tim Skern, Donald B. Smith, Matthew B. Sullivan, Nobuhiro Suzuki, Dann Turner, Koenraad Van Doorslaer, Anne-Mieke Vandamme, Arvind Varsani, Nikos Vasilakis

**Affiliations:** 1 Nuffield Department of Medicine, University of Oxford, Oxford, United Kingdom; 2 Quadram Institute Bioscience, Norwich Research Park, Norwich, United Kingdom; 3 Departamento de Fitopatologia/BIOAGRO, Universidade Federal de Viçosa, Viçosa, Brazil; 4 Structure and Cell Biology of Viruses Lab, Center for Cooperative Research in Biosciences—BRTA, Derio, Spain; 5 Basque Foundation for Science, IKERBASQUE, Bilbao, Spain; 6 Department of Microbiology, Faculty of Science, Mahidol University, Bangkok, Thailand; 7 Departamento de Microbiologia/BIOAGRO, Universidade Federal de Viçosa, Viçosa, Brazil; 8 National Genomics Data Center, Beijing Institute of Genomics, Chinese Academy of Sciences and China National Center for Bioinformation, Beijing, China; 9 University of Chinese Academy of Sciences, Beijing, China; 10 Department of Molecular Virology, Adam Mickiewicz University, Poznan, Poland; 11 Institute of Virology, Charité-Universitätsmedizin Berlin, corporate member of Free University Berlin, Humboldt University, Berlin, Germany; 12 Berlin Institute of Health, Berlin, Germany; 13 Department of Ecology, Evolution and Natural Resources, School of Environmental and Biological Sciences, Rutgers The State University of New Jersey, New Brunswick, New Jersey, United States of America; 14 The Center for Vaccine Research, University of Pittsburgh School of Medicine, University of Pittsburgh, Pittsburgh, Pennsylvania, United States of America; 15 Institute of Biodiversity, Faculty of Biological Sciences, Cluster of Excellence Balance of the Microverse, Friedrich-Schiller-University, Jena, Germany; 16 Theoretical Biology and Bioinformatics, Science for Life, Utrecht University, Utrecht, the Netherlands; 17 Instituto de Biología Integrativa de Sistemas (I2SysBio), CSIC-Universitat de València, Valencia, Spain; 18 Santa Fe Institute, Santa Fe, New Mexico, United States of America; 19 Instituto de Biotecnología y Biología Molecular, CCT-La Plata, CONICET, UNLP, La Plata, Argentina; 20 Department of Biology, University of Oxford, Oxford, United Kingdom; 21 National Center for Biotechnology Information, National Library of Medicine, National Institutes of Health, Bethesda, Maryland, United States of America; 22 Institut Pasteur, Université Paris Cité, CNRS UMR6047, Archaeal Virology Unit, Paris, France; 23 Integrated Research Facility at Fort Detrick (IRF-Frederick), National Institute of Allergy and Infectious Diseases, National Institutes of Health, Fort Detrick, Frederick, Maryland, United States of America; 24 Division of Vector-Borne Diseases, National Center for Emerging and Zoonotic Infectious Diseases, Centers for Disease Control and Prevention, Fort Collins, Colorado, United States of America; 25 Department of Microbiology, University of Alabama at Birmingham, Birmingham, Alabama, United States of America; 26 Institute of Biochemistry and Biophysics of the Polish Academy of Sciences, Warsaw, Poland; 27 Department of Biosystems, KU Leuven, Leuven, Belgium; 28 School of Microbiology and APC Microbiome Ireland, University College Cork, Cork, Ireland; 29 Department of Bioinformatics and Databases, Leibniz Institute DSMZ—German Collection of Microorganisms and Cell Cultures GmbH, Braunschweig, Germany; 30 Division of Molecular and Cellular Biosciences, National Science Foundation, Alexandria, Virginia, United States of America; 31 Molecular and Integrative Biosciences Research Programme, Faculty of Biological and Environmental Sciences, University of Helsinki, Helsinki, Finland; 32 Max Planck Tandem Group in Computational Biology, Departamento de Ciencias Biológicas, Universidad de los Andes, Bogotá, Colombia; 33 MRC-University of Glasgow Centre for Virus Research, Glasgow, United Kingdom; 34 Department of Energy Joint Genome Institute, Lawrence Berkeley National Laboratory, Berkeley, California, United States of America; 35 Istituto per la Protezione Sostenibile delle Piante, CNR, UOS Bari, Bari, Italy; 36 Department of Biochemistry, Molecular Biology, Entomology and Plant Pathology, Mississippi State University, Mississippi State, Mississippi, United States of America; 37 School of Cellular and Molecular Medicine, Faculty of Life Sciences, University of Bristol, Bristol, United Kingdom; 38 Medical University of Vienna, Max Perutz Labs, Vienna Biocenter, Vienna, Austria; 39 Departments of Microbiology and Civil, Environmental, and Geodetic Engineering, Ohio State University, Columbus, Ohio, United States of America; 40 Institute of Plant Science and Resources, Okayama University, Kurashiki, Okayama, Japan; 41 School of Applied Sciences, College of Health, Science and Society, University of the West of England, Bristol, United Kingdom; 42 School of Animal and Comparative Biomedical Sciences, Department of Immunobiology, BIO5 Institute, and University of Arizona Cancer Center, Tucson, Arizona, United States of America; 43 KU Leuven, Department of Microbiology, Immunology and Transplantation, Rega Institute for Medical Research, Leuven, Belgium; 44 Center for Global Health and Tropical Medicine, Instituto de Higiene e Medicina Tropical, Universidade Nova de Lisboa, Lisbon, Portugal; 45 The Biodesign Center for Fundamental and Applied Microbiomics, School of Life Sciences, Center for Evolution and Medicine, Arizona State University, Tempe, Arizona, United States of America; 46 Department of Pathology, Center of Vector-Borne and Zoonotic Diseases, Institute for Human Infection and Immunity and World Reference Center for Emerging Viruses and Arboviruses, The University of Texas Medical Branch, Galveston, Texas, United States of America

## Abstract

A universal taxonomy of viruses is essential for a comprehensive view of the virus world and for communicating the complicated evolutionary relationships among viruses. However, there are major differences in the conceptualisation and approaches to virus classification and nomenclature among virologists, clinicians, agronomists, and other interested parties. Here, we provide recommendations to guide the construction of a coherent and comprehensive virus taxonomy, based on expert scientific consensus. Firstly, assignments of viruses should be congruent with the best attainable reconstruction of their evolutionary histories, i.e., taxa should be monophyletic. This fundamental principle for classification of viruses is currently included in the International Committee on Taxonomy of Viruses (ICTV) code only for the rank of species. Secondly, phenotypic and ecological properties of viruses may inform, but not override, evolutionary relatedness in the placement of ranks. Thirdly, alternative classifications that consider phenotypic attributes, such as being vector-borne (e.g., “arboviruses”), infecting a certain type of host (e.g., “mycoviruses,” “bacteriophages”) or displaying specific pathogenicity (e.g., “human immunodeficiency viruses”), may serve important clinical and regulatory purposes but often create polyphyletic categories that do not reflect evolutionary relationships. Nevertheless, such classifications ought to be maintained if they serve the needs of specific communities or play a practical clinical or regulatory role. However, they should not be considered or called taxonomies. Finally, while an evolution-based framework enables viruses discovered by metagenomics to be incorporated into the ICTV taxonomy, there are essential requirements for quality control of the sequence data used for these assignments. Combined, these four principles will enable future development and expansion of virus taxonomy as the true evolutionary diversity of viruses becomes apparent.

## Abstract 2

Transforming an existing phenotypic classification of viruses into one based on evolutionary relationships that can accommodate the vast number of viruses characterized in metagenomics and environmental studies is an ongoing challenge. This Consensus View explains how such a taxonomy can be expanded to encapsulate viral diversity and to recognize independent biological origins of different virus groups.

## Introduction

The International Committee on Taxonomy of Viruses (ICTV) is the official body mandated by the International Union of Microbiology Societies to develop and maintain a taxonomy of viruses and the naming of their taxa. Throughout its history, the rules and codes associated with taxonomy have been updated many times in response to new discoveries, changes in understanding of evolutionary relationships among viruses, and, importantly, the advent of new technologies, such as high-throughput sequencing (HTS) that have vastly increased our global knowledge of viral diversity.

With roots in a pregenomic age, the criteria used for virus classification (see Box 1 for definitions of terms used in virus taxonomy) and taxon nomenclature were originally and by necessity based on observational properties of virus isolates, including the morphology of virion particles [1], type of nucleic acid in their genomes [2], and physical attributes such as susceptibility to inactivation by high temperature, organic solvents, and low pH [3,4]. While the vast majority of viruses now included in the ICTV taxonomy have been characterized at the genomic level (and this has been recently introduced as prerequisite for classification), there remains active debate on the extent to which historical reliance on physical and biological properties might continue to be useful as classification criteria and, indeed, whether viruses need to be characterized in in vitro culture or by virion visualization to be eligible for taxonomic assignment [5,6]. This topic is hotly debated among virologists, as among prokaryotic and fungal taxonomists, who are discussing whether to require strain isolation, phenotypic characterization, and placement in publicly available collections. Current prokaryote and fungi species lists capture only a small fraction of the true genetic diversity of these organisms in the wider environment, with species totals in the tens of thousands rather than the millions that genomic surveys estimate to exist [7,8].

**Box 1. Definitions of terms used in virus taxonomy**

**Classification**: The process of assigning viruses to groups. This process can be performed on different sets of features leading to different classification schemes. In the ICTV taxonomy, classification is evolutionarily based and hierarchical. The groups are named taxa.

**Nomenclature**: The naming of viruses or taxa. Taxon nomenclature is regulated by the ICTV and has a number of typographical restrictions concerning italicization and capitalization; taxon names above the rank of species possess suffixes to indicate taxonomic rank. Species nomenclature follows a binomial format (genus name + species epithet). In contrast, the naming of viruses is not regulated by the ICTV.

**Rank**: A relative position in a hierarchy. The ICTV taxonomy provides up to 15 ranks, with the highest (top) termed realm, and the lowest (basal) rank termed species.

**Taxon**: A taxonomic category for a group of viruses that is evolutionarily related and whose members may share similar properties. In a hierarchical classification, the demarcation criteria that define higher-rank taxa are shared with all lower-level taxa within.

**Taxonomy**: A biological classification based on evolutionary relationships in which viruses are assigned to a series of hierarchical taxa (classification) with regulated naming of component taxa (nomenclature).

An expert group convened by the ICTV in 2016 debated and affirmed a policy to allow viruses known from their genome sequences alone to be incorporated into virus taxonomy. This policy enables taxonomic assignments without requiring prior knowledge of a virus phenotypic properties, such as host range or pathogenicity, nor isolation of viruses in cell culture/local lesion hosts, or visualization of virions [9]. Subsequent discussions led to the publication of guidelines for minimum standards for virus sequence data to ensure that viruses assigned to the ICTV taxonomy are represented by complete or coding-complete genomic sequences, which are accurately assembled and free from artifacts [10,11]. This development has led to large numbers of new taxa being incorporated into the official taxonomy, primarily from genomic data accrued from large-scale metagenomic surveys [12–18]. It also led to a renewed debate on the merits of having different criteria being used for taxonomic assignments among different groups of viruses. In particular, the emphasis on biological properties for many viruses infecting animals and plants versus the almost exclusive use of nucleic acid–based features for viruses infecting prokaryotes.

The creation of a unified evolutionary taxonomy that incorporates viruses classified both by traditional and metagenomics-based analyses requires considerable knowledge and insight into how virus properties are genomically encoded, about their evolutionary histories, and the influence of past recombination or reassortment of genomic regions on phylogenetic congruence. Furthermore, viruses have multiple, independent, and likely ancient evolutionary origins (reviewed in [15,19,20]). To develop criteria for assigning viruses to taxa, consensus is required on which genes are most informative in recovering relationships that best represent the evolutionary histories of each of these different clades.

### Aims

A group of 45 basic and clinical virologists, bioinformaticians, and evolutionary and structural biologists met in Oxford, United Kingdom, in April 2022, to develop a community-wide consensus on methodologies used for virus classification and to establish an integrated and internally consistent taxonomic framework. The discussions focused primarily on how an evolutionary taxonomy of all viruses infecting eukaryotes, archaea, and bacteria might be constructed, which tools and approaches could be used, and how this process could be guided by identification of the most evolutionarily informative attributes of virus genome sequences and their organization. The group also considered the broader issue of how to reconcile an expanding genetic and structural classification with a partly phenetic classification developed by virologists over many decades that takes into account, among other properties, clinical and regulatory utility, virus/host ecology, and epidemiology.

The meeting achieved a substantial consensus on a range of approaches and challenges for taxonomy development, with all but two of the 45 participants endorsing a series of agreed recommendations in the form of four virus taxonomy principles (Box 2). We believe these will have long-term relevance and practical utility to inform the continued development of a universal virus taxonomy by the ICTV for many years to come.

**Box 2. Recommendations for future virus taxonomy**

**1: Virus taxonomy should reflect the evolutionary history of viruses.** Most viruses can be assigned to independent virus realms, each with an inferred separate evolutionary origin. Members of each realm possess sets of ancestral orthologous genes, termed hallmark genes, typically corresponding to replication or virion formation modules within their genomes. Their evolutionary relationships define monophyletic taxonomic assignments within each of these virus groups.

**2: Virus properties may guide assignment of ranks to maximize their utility.** While evolutionary relationships determine the topology of virus taxonomies, the ranks assigned within it are human-made constructs, with up to 15 available from realm to species. Placement of viruses should follow patterns of evolutionary, genomic, and phenotypic properties; for example, species assignments may be based on host range, disease associations, or epidemiology, provided that such categories result in monophyletic groups.

**3: Taxonomy is but one of many possible means to classify viruses.** The taxonomy produced by the ICTV provides an overarching framework for classifying viruses based on evolutionary relationships. However, alternative classifications based on, for example, clinical or epidemiological properties or regulatory requirements have their own utilities in specific circumstances. These may not follow evolutionary relationships (like the Baltimore classification) or may include polyphyletic categories, such as arboviruses or human immunodeficiency viruses, that have epidemiological or clinical value but cannot be represented within an evolutionary taxonomy.

**4: Taxonomic assignments of viruses inferred from metagenomic sequences require strict sequence quality control.** Sequence-based assignment of a new taxon in the absence of other virus characterization requires it to be both accurate and complete. Published guidelines for minimum information about an uncultivated virus genome for taxonomic assignment have been produced [10].

### Principle 1. Virus taxonomy should reflect the evolutionary history of viruses

**Fig 1:**
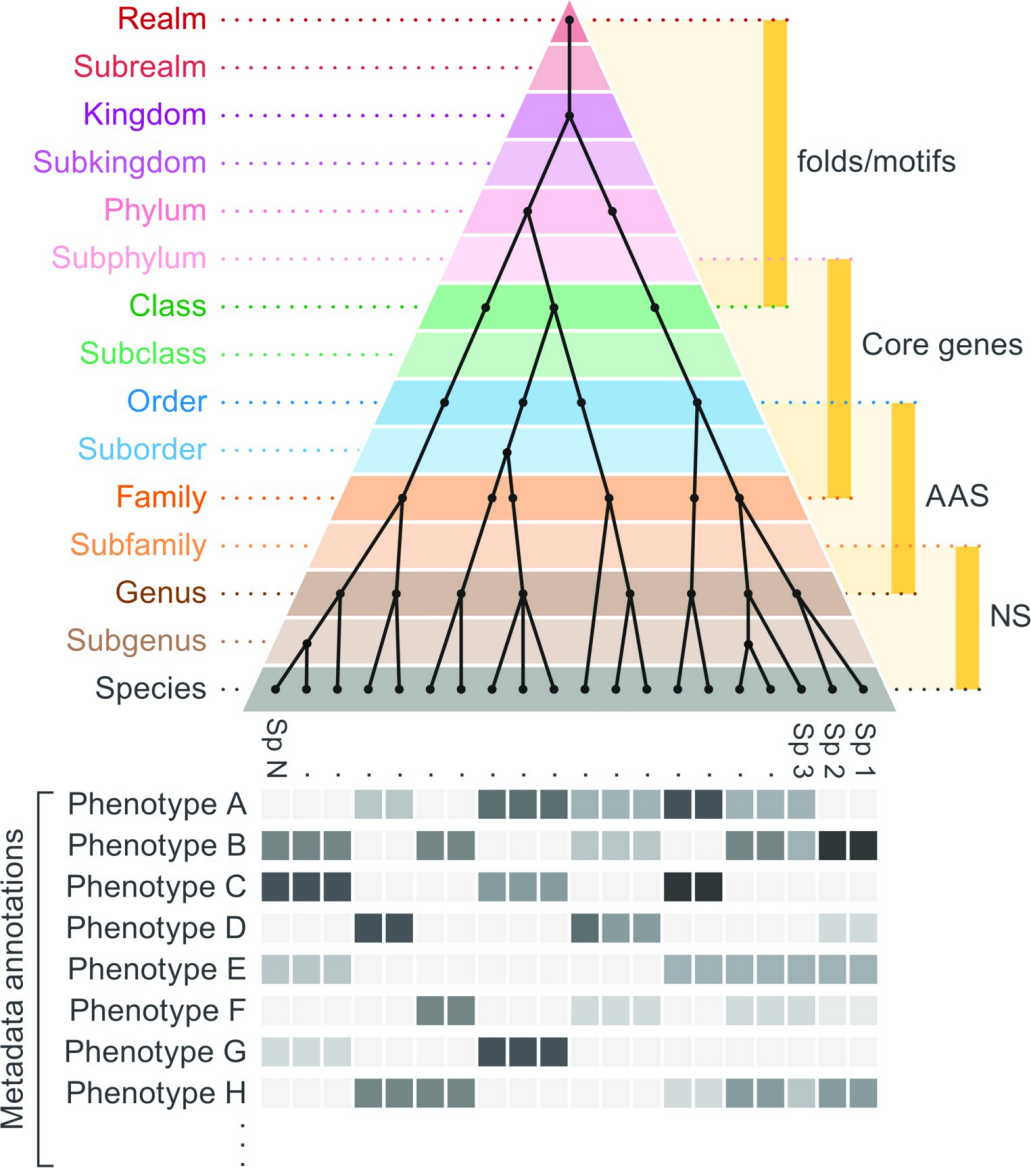
Ranks used in virus taxonomy. Schematic depiction of the 15-rank taxonomic framework used by the ICTV. It includes the methodologies that may be used to determine virus evolutionary relationships and make assignments at each rank. The pyramid shape indicates that the number of taxa increases from the top rank (realm) to the most basal rank (species, Sp.). The names of the 15 ranks are shown on the left of the pyramid, and the methodologies are on the right (AAS, amino acid sequence similarity; NS, nucleotide sequence similarity). The pyramid includes a hypothetical example of the taxonomy of a realm, indicating the number of taxa at each rank (filled circles). The phenotypic properties of classified viruses that may inform rank placements are depicted below the pyramid.

Ranks used for virus taxonomy (realm, kingdom, phylum, class, order, family, genus, and species) must reflect degrees of evolutionary relatedness of the viruses assigned at each rank (Fig 1). This implies that viruses assigned to an individual rank form a monophyletic clade, i.e., all members of a rank share a most recent common ancestor that is distinct from all other evolutionary lineages assigned to the taxonomy despite the impact of gene acquisition, recombination, or reassortment events on genome organizations. This statement may seem obvious, but it is, in fact, the first formal recognition by the ICTV that virus taxonomy should be guided at all ranks by the inference of evolutionary history. This principle provides the necessary route forward for a taxonomy that can incorporate viruses characterized from metagenomics studies.

#### Virus evolutionary histories and choice of hallmark genes

We recognize that the establishment of a coherent virus taxonomy requires a variety of tools and approaches to reconstruct the underlying evolutionary relationships of viruses across their spectrum of diversity. Reconstruction of the deeper evolutionary histories of viruses is particularly challenging due to the lack of conserved genes across all virus genomes. This reflects the growing certainty that viruses have emerged on multiple independent occasions [20–22]. The impossibility of creating a taxonomic structure for all viruses with a single common ancestor contrasts with the biological classification of cellular life forms that possess a set of core genes, such as those encoding ribosomal proteins and ribosomal RNAs. While acknowledging the reticulate nature of the tree of life, these universal genes testify to the shared ancestry of genes present in bacteria, archaea, and eukaryotes linking back to a last universal cellular ancestor (LUCA) and that can be aligned to infer the deepest evolutionary relationships among all domains of cellular life forms [19,23].

Despite the lack of universal virus genes, considerable progress has been made recently in better defining virus groups that share common ancestry [15,24]. The majority of viruses can be assigned to one of several independent realms, each of which is unified through possession of a shared orthologous gene or gene set, termed hallmark gene(s) [15]. Each realm is inferred to represent a distinct, independent origin of its constituent members. Two major functional components, the genome replication module and the virion formation module [25], are currently used for realm definition. These hallmark genes are thus considered to be ancestral to the members of each realm [25].

Virion morphogenesis modules were chosen as the defining characters for DNA viruses with larger genomes and govern assignments into the realms *Adnaviria*, *Duplodnaviria*, and *Varidnaviria*. Viruses in these three realms encode major capsid proteins (MCPs) that are structurally radically different, as well as distinct virion assembly and genome packaging machineries [15,19,26], suggesting independent evolutionary origins. The evolutionary relationships of the genes involved in replication were not considered suitable for defining the realms of large DNA viruses because even relatively closely related viruses within the same realm often have distinct genome replication modules. For example, related viruses can encode nonhomologous or distantly related DNA polymerase genes of families A, B, or C that are interspersed with cellular counterparts. Some may lack DNA polymerase genes altogether and instead encode diverse replication initiators that facilitate the recruitment of the host replisome [25,27].

On the other hand, the key features of the genome replication machinery are the most suitable for defining the realms *Riboviria* [15], *Monodnaviria* (ICTV Taxonomy proposal 2019.005G.R.Monodnaviria), and *Ribozyviria* (ICTV Taxonomy proposal 2020.012D.R.Ribozyviria). The realm *Riboviria* unifies RNA viruses (kingdom *Orthornavirae*) and reverse-transcribing viruses (kingdom *Pararnavirae*), all of which encode homologous right-handed palm-domain RNA-directed RNA polymerase (RdRP) or reverse transcriptase (RT) genes, respectively. The phylogeny of these RdRPs and RTs was, therefore, used to guide the taxonomy within the *Riboviria*. In contrast, the capsid genes of RNA viruses fall into several unrelated groups, many likely to have been separately acquired from their hosts [28] or are completely absent. Analogously, all members of the realm *Monodnaviria* encode homologous histidine–hydrophobic residue–histidine (HUH) superfamily endonucleases [15,29], but the virion morphogenesis modules are distinct for viruses from different phyla within this realm. Finally, members of the *Kolmioviridae*, currently the sole family in the realm *Ribozyviria*, have small circular negative-sense RNA genomes that do not encode an RNA polymerase but contain a particular ribozyme that serves to define the realm.

#### Modular evolution of viruses

Virus evolution is frequently punctuated by large-scale genome reorganizations and the exchange of gene modules analogous to horizontal gene transfer in prokaryotes. For example, alpha-, beta-, gamma-, and deltaflexiviruses and tymoviruses possess an evolutionarily conserved set of replication genes (Rep) that define their classification in the order *Tymovirales* in the realm *Riboviria*. However, their capsid morphologies are diverse, including particles that are isometric (members of the *Tymoviridae*), filamentous/helical (viruses in the *Alphaflexiviridae*, *Betaflexiviridae*, and *Gammaflexiviridae*), or form no particles at all (members of the *Deltaflexiviridae* and fungus-infecting members of the *Alphaflexiviridae*). Even within a family, the phylogeny of capsid genes may be noncongruent with that of the replication genes, such as between genera of *Alphaflexiviridae* [30]. Similarly, members of the order *Martellivirales* share relatively closely related RdRPs and other genes involved in replication, such as helicases and capping enzymes, but produce flexible filamentous, rod-shaped, or icosahedral particles constructed from unrelated capsid proteins [28], or no classic virions at all (i.e., endornaviruses), suggesting the acquisition or loss of capsid morphogenesis genome modules from taxonomically distant viruses.

Furthermore, capsid genes can be exchanged between viruses that are otherwise evolutionarily unrelated. For example, a range of plant and animal RNA viruses and small single-stranded (ss) DNA viruses encode homologous horizontal single a jelly-roll capsid proteins, despite the RNA viruses being assigned to the realm *Riboviria* and the ssDNA viruses to the realm *Monodnaviria* [31–33]. Some prokaryotic viruses, in particular those alternating between lysogenic and lytic infections (“temperate” viruses), such as λ-like phages and those in the realm *Duplodnaviria* infecting *Mycolicibacterium* species, are substantially influenced by horizontal gene transfer [34]. These viruses possess a so-called mosaic genome structure, in which different parts of the genome can have quite different evolutionary histories [34]. In such cases, the placement of taxonomic boundaries to form monophyletic groups at certain ranks is arbitrary as there are multiple possible evolutionary histories.

Although gene-sharing networks are informative for tracking gene exchange across virus groups [35], the relationships they depict violate the principles of ancestral descent that are used in taxonomy. Therefore, while different gene components are equally parts of the evolutionary histories of viruses and contribute to their phenotypes, for pragmatic purposes, we assign primacy to the most evolutionarily conserved hallmark genes in the construction of a hierarchical taxonomy. The use of hallmark genes for virus taxonomy is conceptually analogous to the use of a core set of conserved genes (primarily those for translation system components) for taxonomy of cellular life forms and eschews the use of the much more variable complements of genes subjected to horizontal gene transfer and loss [36]. Alternative taxonomies could be developed by selection of different genes to determine relatedness (for example, through basing the taxonomy of RNA viruses on capsid gene relationships, or of large DNA viruses by DNA polymerase genes). However, these typically yield a much greater number of unrelated virus groups and a less parsimonious association with virus properties.

#### Methodology for virus phylogenetics and taxonomy

Within individual virus realms, currently, a range of genome sequence comparison methods are needed to describe and assign viruses to different taxonomic ranks. For viruses with similar genome sequences, i.e., within the same species and genus, genetic relationships may be inferred from alignments of nucleotide or amino acid sequences of (near) complete genomes or of specific genes. The relationship among viruses can be further explored by phylogenetic tree inference and analysis, and where this is not practical, clustering by sequence similarity and analysis of pairwise distance distributions using tools such as PASC [37], DEmARC [38], and VIRIDIC [39]. However, these values only serve as an approximation of evolutionary relatedness [40,41]. The latter may be better inferred by phylogenetic methods that are also capable of calculating clade support, such as VICTOR [42] (Table 1).

**Table 1:** Examples of methodologies used for virus classification at different taxonomic ranks*.

Method	Principle	Rank range	Ref
DEmARC	Analysis of distributions of pairwise evolutionary distances between nucleotide or amino acid sequences	Suborder, Family, Subfamily, Genus, Subgenus, Species	[38]
GRAViTy	Virus relationships from composite Jaccard distances between HMM profiles and genome organizational models	Order, Family, (Genus)	[46]
HSF	Identification of structural equivalence and calculation of structural distances for structure-based phylogenetics	Realm, Kingdom, Phylum, Class, Order, Family, Genus	[58,68]
PASC	Analysis of pairwise nucleotide sequence distance distributions	Genus, Species	[37]
PhageClouds	Graph database of phage genomic sequences and intergenomic distances	Subfamily, Genus, Species	[69]
SDT	Pairwise nucleotide sequence alignment and identity calculation	Species	[70]
vConTACT2	Whole-genome gene sharing profiles integrating hierarchical clustering and confidence scores	Order, Family, Subfamily, Genus	[44,45]
VICTOR	Phylogenomic method optimized to ICTV classification that reports both sequence identity- and gene content-based phylogenies along with a suggested classification; works with either nucleotide or amino acid datasets	Family, Subfamily, Genus, Species	[42]
ViPTree	Virus relationships from genomic distances based translated nucleotide scores using tBLASTx	Family, Subfamily, Genus	[49]
VirClust	Hierarchical clustering based on core protein analysis	Order, Family, Subfamily	[71]
VIRIDIC	Calculates intergenomic similarities between pairs of viral genomes based on BLASTN alignments	Family, Genus, Species	[39]

*Discussions of tools dedicated to general reconstruction of phylogeny based on multiple sequence alignments are beyond the scope of this paper (for more information about this subject, see [72,73]). A more extensive list of virus bioinformatics tools including tools for virus taxonomy can be found at https://evirusbioinfc.notion.site/evirusbioinfc/18e21bc49827484b8a2f84463cb40b8d?v=92e7eb6703be4720abf17a901bc9a947.

At the intermediate levels of family, order, and class, relationships can be inferred by comparing sequences of evolutionarily conserved hallmark genes using sensitive methods for protein family profile comparison, such as HHPred [43], with subsequent phylogenetic analysis using appropriate methods for tree inference, for example, maximum likelihood methods. Comparison of hallmark proteins can be combined with metrics based on gene content, gene order and orientation (synteny), and other aspects of genome organization, using tools such as vConTACT2 [44,45] and GRAViTy [46], which are based on hierarchical clustering of gene sharing networks and the detection of hidden Markov model profiles of conserved protein families, with GRAViTy also taking into account metrics based on gene order and genome organization [47,48]. ViPTree [49] has been also used to define family level taxa of prokaryotic double-stranded (ds) DNA viruses, whereas VICTOR [42] can classify all prokaryotic viruses at the species, genus, subfamily, and family ranks through a joint clustering- and phylogeny-driven approach.

Taxonomic assignments at higher ranks, such as phyla, kingdoms, and realms, are based either on sequence comparison of the most highly conserved hallmark proteins and/or on protein structure comparisons. The latter can be informative for making evolutionary comparisons because homologous proteins typically retain similar structures, even when the corresponding amino acid sequences have diverged to the point that they are no longer sufficiently similar to infer homology based on sequence alone. Structure-based comparison methods include clustering based on estimates of distances between structures and structure-based phylogenetic analysis [50]. Much of the data used for this purpose originates from structures resolved experimentally with X-ray crystallography and, more recently, cryo-electron microscopy [51,52]. However, protein structure prediction methods have become much more accurate and insightful with the potential to enable large-scale bioinformatics-based reconstructions of structural features from sequence data alone. An important caveat is that, at this time, the recently developed and highly successful programs AlphaFold [53] and RosettaFold [54] generalize from known protein structures in the protein databank (PDB), a dataset in which virus proteins are substantially underrepresented [55], thus limiting their predictive power for analysis of relationships among viruses.

Hallmark gene-based assignments at the levels of kingdom and realm can be hampered by high levels of protein sequence divergence, with homology only detectable once high-resolution structures for the corresponding proteins become available. For this reason, the validity of the phylogenetic analyses used to designate kingdoms and phyla through evolutionary relationships among RdRP and RT genes of *Riboviria* using sequence analysis alone has been questioned, as these are based on the purported arbitrariness of the alignment of highly divergent sequences [56]. Alignment methodologies continue to be refined [57], but, ultimately, a range of sequence and protein structure comparison methods are likely to be required to delineate the higher ranks with confidence. Indeed, while protein structure can be influenced by environmental conditions (such as temperature, ionic strength, etc.), the optimal fold determined under standardized conditions is a highly evolutionarily conserved attribute of a protein coding sequence, and structural homology may be recoverable even when detectable sequence homology is lost. Encouragingly, a phylogeny based on protein structure comparisons of the viral RdRPs of members of *Riboviria* [58] matched the relationships inferred by aligned sequence comparison methods [15,59] at all but the highest ranks, as well as by the known functional diversification of these enzymes (i.e., transcription and priming mechanisms) and replication complex morphology [58,60].

**Fig 2:**
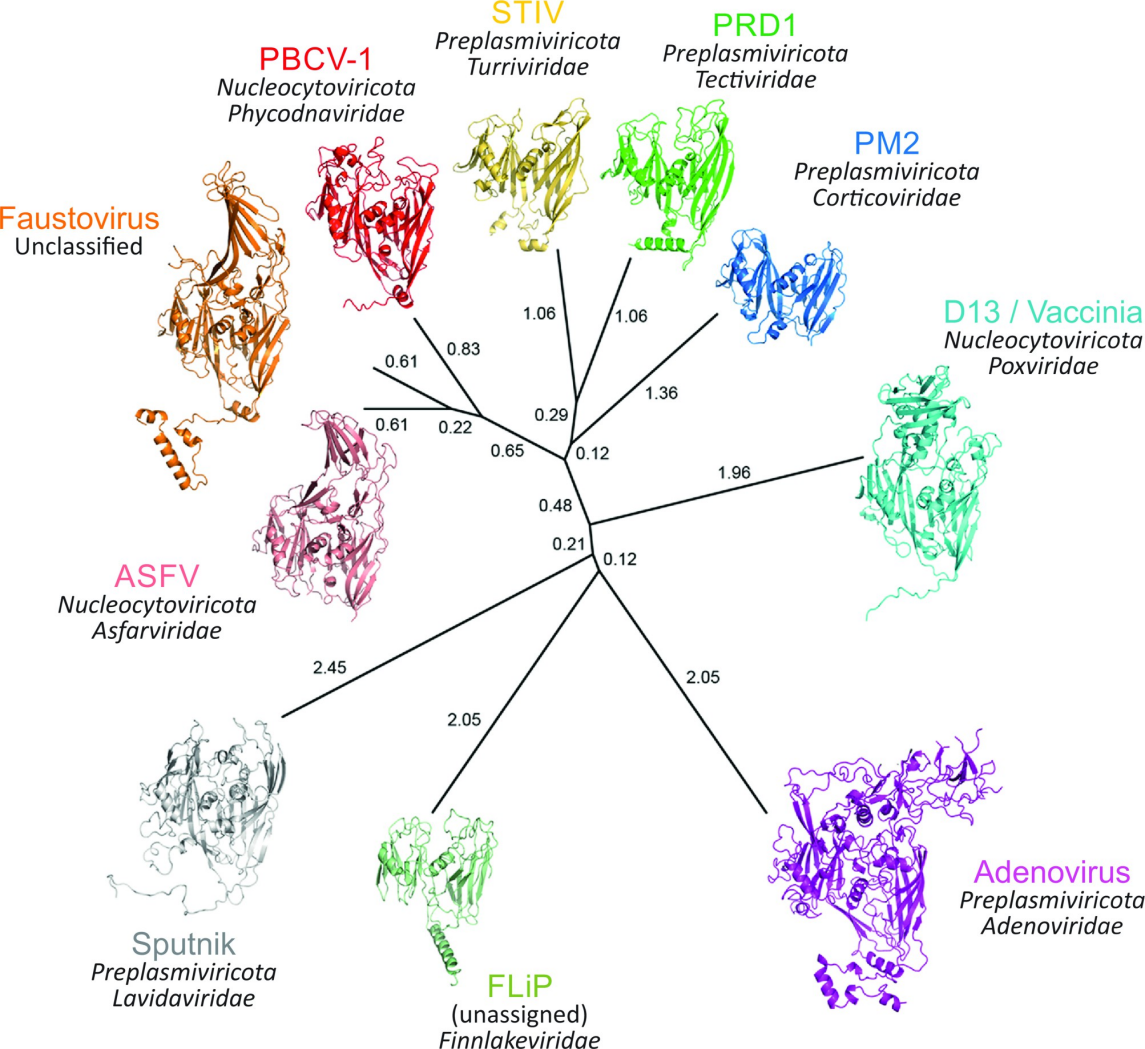
Structure-based dendrogram of capsid proteins of members of the kingdom *Bamfordvirae*. Structure-based phylogenetic tree inferred from major capsid protein (MCP) structures of the members of the kingdom *Bamfordvirae* in the *Varidnaviria* realm. Members of *Bamfordvirae* encode a vertical double-jelly roll fold MCP, which is the hallmark protein of this group of viruses. Next to each MCP structure are the virus name (top), the phylum (middle), and family (bottom), with “Faustovirus” not yet officially classified and *Finnlakeviridae* not yet assigned to any higher taxon. The evolutionary distances across the depicted members of the originally called PRD1-adenovirus viral lineage [67] were calculated with the Homologous Structure Finder software [50] and depicted with PHYLIP (https://evolution.genetics.washington.edu/phylip.html); the evolutionary distances are shown next to each branch. The protein data bank identifiers (PDBid) for the structures are as follows: PRD1: PDBid 1HX6; PBCV-1: 1M3Y; adenovirus: 1P2Z; STIV: 2BBD; Vaccinia D13: 2YGB; Sputnik: 3J26; Faustovirus: 5J7O; FLiP: 5OAC; ASFV p72: 6KU9; PM2: 2W0C. Adapted from [62].

Along similar lines, structure-based clustering and phylogeny of capsid proteins can provide a powerful approach when the reliable inference of evolutionary relationships by sequence comparisons (“traceability”) is lost [61–63]. Thus, deeper evolutionary relationships that underpin capsid protein structure and virion architecture may be used to classify large DNA viruses into realms and kingdoms. As an example, the structure-based PRD1-adenovirus lineage, whose members encode MCPs with a vertical double jelly-roll fold [61,63] (Fig 2), can be assigned to the kingdom *Bamfordvirae*, which falls within the realm *Varidnaviria*. Conversely, established structural relationships can now be used to inform sequence alignments and allow the incorporation of the ever-expanding wealth of virus sequence data into taxonomy [59,64]. Detection of subtle sequence conservation among structurally similar major capsid proteins of large DNA viruses further validates the use of these proteins as hallmarks for *Varidnaviria* and *Duplodnaviria* [64–66].

The ranges of sequence divergence (and, consequently, rank levels) over which the various analytical methods used in virus taxonomy are defined overlap substantially (Table 1). The recent delineation and assignment of a new family of bacterial viruses (*Herelleviridae*) [48] is an illustrative example of the value of such a combined approach. Concordance between multiple methods using different approaches increases the reliability of the taxonomic placement of novel taxa, whereas conflicts are informative regarding both the suitability of different comparison methods, and the nature of the relationships among viruses. Such conflicts can also arise from gene sharing networks and have led to several examples for which ICTV taxonomic revisions were needed [45]. Conflicts between different methods may also indicate the need to postpone taxonomic assignments until more data become available.

#### A six-realm taxonomy of viruses?

Our understanding of virus origins and the evolutionary relationships inferred from hallmark gene trees within realms may change over time as analytical methods improve and new data become available. These may necessitate revisions to virus taxonomy. The extent to which the currently assigned realms encapsulate the full range of virus diversity and their distinct evolutionary origins remains under intense scrutiny. On the one hand, the possibility exists that large-scale metagenomics-based analyses of viruses in the environment are already approaching saturation of higher taxonomic ranks, such that the overall structure of virus taxonomy is stabilizing, even if many taxa remain to be delineated at lower levels. For example, a recent analysis of RNA virus diversity in the marine virome has vastly expanded the number of distinct viruses and putative genera and families, but these mostly can be assigned to the five previously established phyla within the riboviriad kingdom *Orthornavirae* [13]. On the other hand, the recent description of a plethora of RNA viruses sampled throughout the Global Oceans suggests the need to establish at least five additional phyla of RNA viruses [74] and urges some caution in these conclusions, particularly in light of the paucity of studies of virus diversity in other environments.

As an indication of future possible changes, structural analysis of virus capsid proteins within the realm *Varidnaviria* [75] indicates that this realm is not monophyletic and likely has to be split into two realms corresponding to the current kingdoms *Bamfordvirae* and *Helvetiavirae*. Furthermore, many (relatively) narrow groups of viruses, particularly those with hyperthermophilic archaea as hosts [76], cannot be classified into any of the six realms described to date. It appears highly likely that further characterization of the diversity of these virus groups and their conserved structural and genomic features will lead to the delineation of additional, comparatively small realms and the associated expansion of taxonomic ranks within these taxa [77].

While undoubtedly incomplete, the creation of the rank of realm and the recognition of a separate origin for each provides a substantive basis for a coherent and stable taxonomy of the viruses within them. The high value of hallmark gene relationships should be at the core of classification decisions at higher taxonomic ranks and will provide a blueprint for realm expansion, as needed in the future.

### Principle 2. Virus properties may guide the assignment of ranks to maximize their utility

The primary value of taxonomy lies in its ability to sort organisms into categories that reflect their evolutionary history, be it at the species, genus, or family level, or higher ranks. All evolutionarily based taxonomic codes, including that of the ICTV, follow the principle that each rank must be congruent with evolutionary relationships. The ICTV Code states that species should be monophyletic and, therefore, cannot group unrelated viruses sharing similarities in their physical properties, type of host/vector, disease associations, or other aspects of their phenotype, which might be polyphyletic in nature. This stipulation conflicts with many alternative (nontaxonomic) classifications of viruses used in medical, veterinary, agricultural, and regulatory fields described in Principle 3.

#### Assignment of taxonomic ranks

A hierarchical taxonomy based on evolutionary relationships of hallmark genes is inviolate and cannot support polyphyletic categories. However, the taxonomic rank is ultimately a human-made construct that arbitrarily assigns diversity into discrete categories that can be more readily conceptualized and named. The ICTV provides up to 15 ranks to partition virus diversity, from realm to species [78], although most viruses have historically been assigned to a more limited range, typically family, genus, and species. The placement of viruses at lower ranks of the taxonomy should follow patterns of natural clustering; virological knowledge and judgment are required to ensure, as far as possible, that placements also create informative categories for viruses with known phenotypic properties. Species assignments might therefore divide viruses based on their host range, disease associations, and epidemiological distributions, provided that such groups of viruses are monophyletic. The number of thresholds for delineating taxa at a given rank in the various virus groups would ideally be minimized to yield a more uniform taxonomy, although differences will remain, for example, between DNA and RNA viruses, which, in general, evolve at very different rates.

Such choices and the delineation of associated sequence divergence thresholds are typically made by expert groups of virologists, in most cases, ICTV Study Groups. As an example, the various genotypes of hepatitis C virus (HCV), all of which exclusively infect humans, were assigned by the ICTV *Flaviviridae* Study Group to the species *Hepacivirus C*, in distinction from those infecting New World monkey species (*Hepacivirus A* and *Hepacivirus B*) and horses (*Hepacivirus D*) [79]. This is possible because each group of host-associated viruses is monophyletic. The Study Group did not consider HCV genotypes, themselves each forming monophyletic groups, to represent separate species because they were not thought to be sufficiently different clinically or epidemiologically to merit such an assignment. This is despite their nucleotide sequence divergence (approximately 30%) being comparable to that between members of species assigned to other *Flaviviridae* genera, such as *Pestivirus A*, *Pestivirus B*, *Pestivirus C*, and *Pestivirus D*, which, in this case, show substantially different host ranges and disease associations. This constitutes one of many cases in which ecological drivers (in this case, host range) have shaped the evolutionary history of viruses and can be used to inform taxonomic outcomes.

Similarly, virological knowledge informed a revision of species demarcation criteria among members of the genus *Orthobunyavirus* (family *Peribunyaviridae*) [80]. The different geographical distributions, vector and host associations, and pathogenicities of Bunyamwera virus, Batai virus, Cache Valley virus, Ngari virus, Potosi virus, and Tensaw virus, all of which were originally assigned to a single species *Bunyamwera orthobunyavirus*, were considered to render these viruses so phenotypically distinctive as to warrant assignment to separate species [81]. The species nucleotide similarity thresholds demarcating species were accordingly increased to ensure that each of these distinctive viruses were assigned to different species, resulting in the reclassification of species within the genus (ICTV taxonomy proposal 2018.008M.A.v1.Orthobunyavirus_38sp).

The “usefulness” of a taxonomy combining clustering by sequence similarity with phenotypic properties highlights the need for extensive interdisciplinary work bridging the fields of bioinformatics and virology, creating and maintaining taxonomy as the knowledge of viral diversity and their disease impact increases. At higher ranks, evolutionary and structural biologists may provide the more sophisticated approaches required to extract and evaluate genome sequence and structural features that depict the deepest evolutionary relationships of virus kingdoms and phyla.

#### Assessment of taxonomic ranks to viruses discovered in metagenomic studies

For viruses identified by genomes assembled in metagenomic sequence analyses, a phylogeny-based classification is required. Although information on the host range, effects on the host, and other phenotypic traits of these viruses is typically lacking, many of their attributes might be inferred from their genome organization and composition, evolutionary affinities of different genes, and base composition. In some cases, these characteristics may also suggest the likely host range [82–84]. Additional indications of the potential host range for these viruses can also be derived from their co-occurrence with specific groups of potential host organisms, as well as matches to CRISPR spacers in the case of viruses infecting bacteria and archaea [85–88]. As a follow-up to metagenomics, the properties of individual proteins or whole viruses can be experimentally determined through reverse genetics and characterization in vitro and in vivo, when possible (e.g., [89]). Nevertheless, in the (near) absence of phenotypic information, the taxonomy of these viruses, especially at the ranks below family, presents a challenge that might not be fully overcome until the genome analysis is complemented by virological studies at some point in the future.

There is, therefore, an asymmetry between the classification and rank assignments of viruses with well-defined phenotypic properties and the entirely genome-based classification required for viruses characterized by metagenomic analyses alone. Nevertheless, there is a clear consensus that viruses identified in metagenomic studies should be incorporated by the ICTV into virus taxonomy as far as the evidence allows [9]. Indeed, at this time and for the foreseeable future, this route provides by far the greatest source of information on genomic diversity of viruses, and recent metagenomics studies have led to the discovery of numerous new groups of viruses infecting hosts belonging to all three domains of cellular life. This has been a crucial advance in virology, and we must ensure that such viruses are incorporated into a taxonomy that will eventually catalog the true extent and complexity of the virosphere.

### Principle 3. An evolutionary taxonomy is but one of many possible means to classify viruses

We recognize that classifications of viruses by clinicians, veterinarians, agronomists, and regulators may differ from a virus taxonomy that is grounded in evolutionary relationships. Numerous widely used clinical or veterinary virus designations cannot be supported by taxonomic assignments but often better serve clinical and regulatory purposes. The following cases, drawn from many possible examples, illustrate the various forms of mismatch that can occur.

#### Polyphyletic groups of viruses

**Fig 3:**
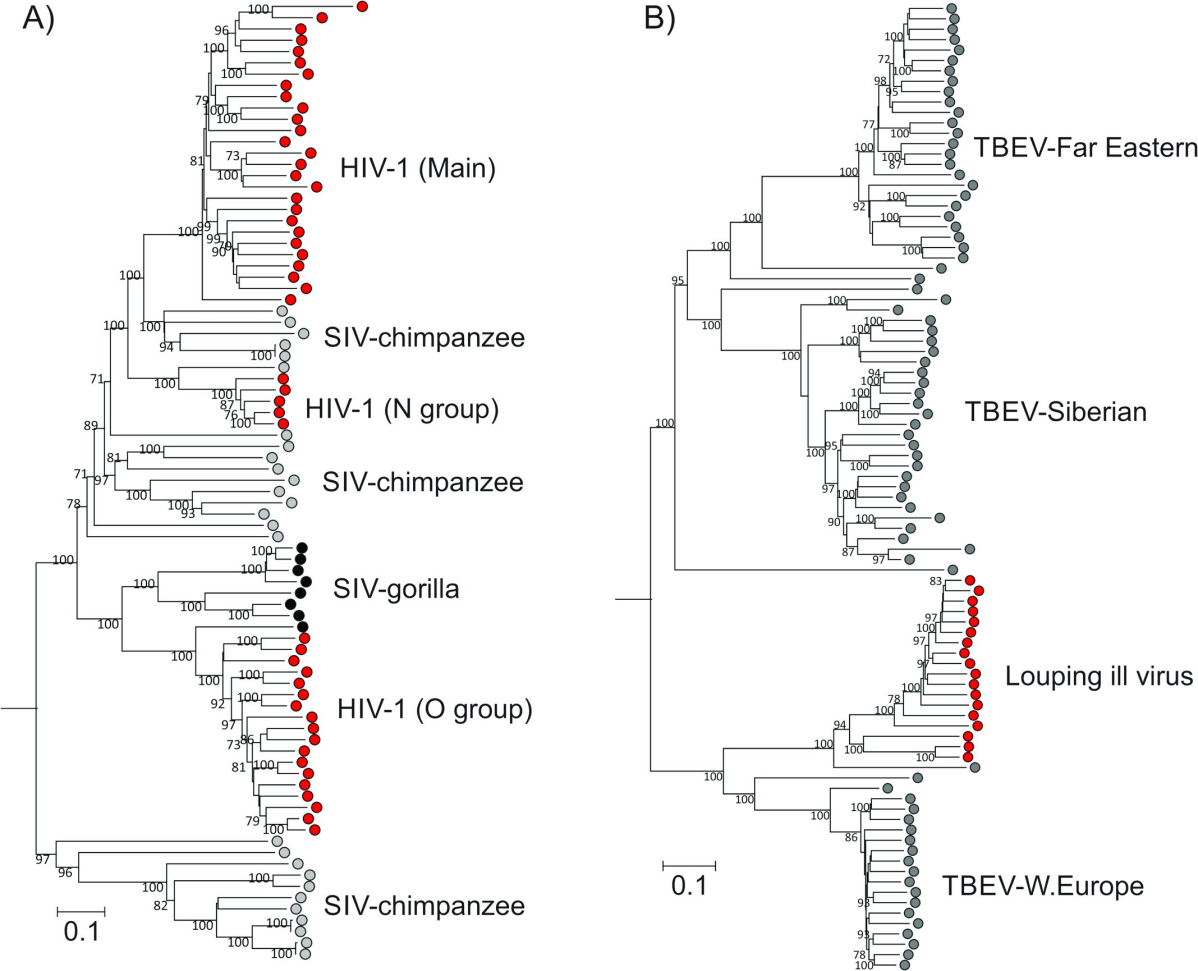
Examples of incompatibilities between species assignments and phylogenetic groupings. (**A**) Genetic relationships of HIV-1 (red dots) with simian immunodeficiency viruses infecting chimpanzees (gray dots) and gorillas (black dots). HIV-1 strains are polyphyletic and cannot be assigned to a single species taxon without incorporating nonhuman viruses within the definition. (**B**) Genetic relationships of louping ill virus (LIV) with tick-borne encephalitis virus (TBEV) strains isolated in Europe and Asia, with the principal groups labeled. Although LIV (red dots) is assigned to the species *Louping ill virus*, it lies within the phylogenetic tree created by strains of TBEV that all belong to the species *Tick-borne encephalitis virus*. The current assignment of LIV as a species therefore logically prevents strains of TBEV being assigned into a single species if species were to remain monophyletic. Trees were constructed from maximum composite likelihood distances between nucleotide sequences of (A) the *pol* gene of HIV-1/SIV and (B) the complete coding sequence of TBEV and LIV. To investigate the robustness of branches, nucleotide positions were bootstrap resampled 100 times as implemented in the MEGA7 program [91]; branches with 70% or greater support are labeled. The HIV-1/SIV tree was rooted using the HIV-2 sequence, M31113; the TBEV/LIV tree was rooted using the closely related Omsk haemorrhagic fever virus sequence, AY193805. Both trees have been annotated with a scale bar indicating substitutions per site.

The clinical and societal utility of the terms human immunodeficiency virus type 1 (HIV-1) and HIV type 2 (HIV-2) as the causative agents of AIDS requires no further explanation. However, the taxonomic assignment of these viruses has remained problematic because neither possesses a single common ancestor distinct from chimpanzee- (HIV-1) or sooty mangabey- (HIV-2) infecting viruses from which they derive (the phylogeny of HIV-1 is shown in Fig 3A) [90]. The only consistent ways to incorporate these viruses into a virus taxonomy based on evolutionary relationships are either to assign them as members of two species that each include lentiviruses infecting apes or Old World monkeys, or to designate each clade of HIV-1 (groups M, N, and O) and HIV-2 (groups A, B, P, and others) as separate species, these being distinct from multiple additional species of simian viruses. However, neither of these potential taxonomies creates species that map directly onto the terms HIV-1 and HIV-2 or the collective term HIV. This discrepancy illustrates the need for separate and parallel classification of HIVs in clinical usage, distinct from their taxonomic classifications at the species level in the genus *Lentivirus*.

There is a parallel with the nomenclature and species assignments of SARS coronavirus (SARS-CoV) and SARS-CoV-2, the latter being the causative agent of COVID-19. As with HIV-1 and HIV-2, however, SARS-CoV and SARS-CoV-2 cannot logically be assigned to separate virus species despite their clinical distinctiveness. They are classified collectively as members of the species *Severe acute respiratory syndrome-related virus*, along with a number of genetically closely sarbecoviruses infecting bats in South-East Asia [92]. While shared species membership follows from their genetic relatedness and inferred evolutionary origins, this taxonomic classification is incongruent with how the medical and wider community might want to classify them as agents of emerging and pandemic human infectious diseases.

#### Viruses that are assigned to different species but are not phylogenetically distinct

Louping ill virus (LIV) and tick-borne encephalitis virus (TBEV) are currently members of two separate species, *Louping ill virus* and *Tick-borne encephalitis virus*, respectively, in the genus *Flavivirus* of the family *Flaviviridae* [68]. The two viruses show distinct geographical distributions and host ranges; LIV is primarily present in sheep and grouse in the uplands of Scotland and South-West England and spread by the deer/sheep tick *Ixodes ricinus*, while TBEV is found in central Europe, Scandinavia, and large parts of Asia, with deer as the primary host reservoir. However, the current taxonomic assignments of LIV and TEBV to two separate species are not supported by their genetic relationships; members of the species *Louping ill virus* are phylogenetically interspersed with members of the species *Tick-borne encephalitis virus*. Consistent with previous observations [93], while sequences of LIVs are monophyletic, their common ancestor is not distinct from that of TBEV strains (Fig 3B), requiring either the assignment of both TBEV and LIV to a single species or the assignment of TBEV to several different species to reflect their distinct evolutionary histories. In neither case would these evolutionary taxonomic assignments reflect current widely used medical and veterinary terminology.

#### Members of the same species with distinct properties

The species *Enterovirus C*, in the family *Picornaviridae*, includes a clinically highly diverse range of member viruses, such as poliovirus types 1, 2, and 3, as well as several largely nonpathogenic enterovirus types. Their assignment to the same species was necessitated by their high degree of sequence similarity and their ability to recombine [94]. However, the poliovirus-associated neuroinvasive phenotype ultimately derives from a difference in the receptors used by these viruses, which is caused by only a handful of amino acid substitutions in the gene encoding the capsid protein VP1. Even though polioviruses are a fundamental element in disease descriptions (e.g., paralytic poliomyelitis and acute flaccid myelitis) and are targets of a largely successful global vaccination campaign, by evolutionary criteria, they cannot be classified into a species separate from many other enteroviruses, however appropriately that might reflect their clinical properties.

#### Virus groups described by phenetic attributes often do not map directly to taxa

Some broader terms such as “respiratory viruses,” “viral meningitis,” and “arboviruses” (arthropod-borne viruses) have wide clinical utility, being the staples of textbooks on infectious diseases and of medical reviews. However, each of the listed groups contains large sets of otherwise unrelated viruses across many different virus families and orders [95].

#### The Baltimore classes are incongruent with virus taxonomy

The classic paper by David Baltimore [96] proposed a classification of viruses based on the types of nucleic acids comprising the viral genome and the strategies used for genome replication and production of mRNA. The seven Baltimore classes, as they became known, have been widely adopted as an informal classification system for viruses. Although this classification system is logical and useful for understanding virus replication, it is at wide variance with evolutionary relationships of the viruses it classifies. Viruses in the realms *Adnaviria*, *Duplodnaviria*, most members of *Varidnaviria*, and some of *Monodnaviria* belong to Baltimore class I (dsDNA genomes), but other viruses in the latter two realms possess ssDNA genomes and accordingly belong to class II. Members of *Riboviria* with RNA genomes are represented in all the remaining classes III to VII, whereas the one member of the *Ribozyviria* is in class V [97].

#### Alternative classification of viruses

The broader point to be drawn from these examples is that an evolutionary taxonomy is not the only way to classify viruses, and its requirement to be congruent with evolutionary relationships can clash with classifications of viruses that are of greater value to clinicians, veterinarians, agronomists, regulators, and other stakeholders. It is similarly important to recognize that although species assignment thresholds can be selected so as to divide viruses into informative categories (see previous section), the requirement for congruency with evolutionary relationships means that this is not always possible.

The assignment of virus species with members often possessing quite distinct clinical or epidemiological attributes contrasts strongly with assignments of bacterial species in the classification of prokaryotes, for which each clinically or otherwise phenotypically distinct bacterial strain has been assigned to separate species with descriptive definitions. The situation is not unlike the historical classification practices of plant virologists; viruses were named after their specific disease presentations and assigned as unique members of a species bearing the same name. For example, a potyvirus causing mosaic in common bean was classified as a member of the species *Bean common mosaic virus*, whereas a second potyvirus causing mosaic in cowpea was classified as a member of *Blackeye cowpea mosaic virus*, and a third potyvirus causing dwarfing (growth reduction) in peanuts was classified as a member of *Peanut stripe virus*. Only when the genomes of these three viruses were sequenced did it become clear that they were closely related and therefore members of the same species, which currently retains the name *Bean common mosaic virus* [98].

#### Virus and species names

The ICTV has always maintained a typographic distinction between virus names and names of the taxa to which they are assigned [99], similar to how many other organisms have names distinct from the names of the species they are assigned to (e.g., humans ↔ *Homo sapiens*). Virus names are simply what virologists want to call viruses, inasmuch as naming practices have also evolved to address concerns such as discrimination and stigmatization (for example, by avoiding names based on geographical locations). Virus names are not within the remit of the ICTV. Viruses can be named with no restrictions on orthography (other than being non-italicized), numbering or language—indeed, many viruses have different names in different languages. Taxon names, in contrast, are within the mandate of the ICTV. They are written with an initial capital letter, are italicized, and may only contain letters of the Latin alphabet, Arabic numerals, and a limited number of symbols. Furthermore, taxon names are constant irrespective of the language that refers to them.

The ICTV typographic conventions reinforce the typological distinction between viruses as real-world objects and taxa as human-made classes or categories. This practice enables virus isolates to be mapped onto species assignments in a much more flexible way than the simple one-to-one correspondence that applies in many other areas of biology. This flexibility provides the framework for an evolutionarily based taxonomy to run alongside a variety of functional virus classifications without conflict. For example, the codes of practice for laboratory handling of viral pathogens are more useful if based upon a categorization of viruses, not species. This is exemplified by specific biocontainment requirements for HIV-1 and HIV-2, and the now highly restrictive regulatory framework established for poliovirus laboratory handling, which contrasts markedly with containment requirements for other members of the species *Enterovirus C*. Far Eastern and Russian spring–summer strains of TBEV are handled at Biosafety Level (BSL) 4 in the United States, whereas the European strains are at BSL 3 and LIV at BSL 2, regulatory distinctions that do not map onto their current species assignments.

Taxonomic definitions can incorporate elements of virus descriptions in their formulations, as is often the case with plant viruses. Similarly, virus descriptions can be more informative if they refer to the corresponding taxa to which they are assigned. Alternative classification systems have their own rules and utilities in the real world, and their use removes conflicts that might otherwise arise if all virus classifications were irrevocably tied to an evolutionarily based taxonomy.

### Principle 4. Taxonomic assignments of viruses inferred from metagenomic sequences require strict sequence quality control

As emphasized above, the assignment of viruses discovered by metagenomic analyses to new or established virus taxa must be based upon their genome sequences. Although the sample source is usually known, a genome sequence provides the key information about relatedness to other viruses, genome organization, inferred mechanisms of replication, and, increasingly, aspects of its virion structure, morphology, and even receptor use. As a unique source of information on the organism that is being classified, the genome sequence accordingly should be coding complete, i.e., contain the entire complement of protein-coding genes of the virus (as far as can be reasonably inferred), annotated and effectively free from sequencing or assembly errors. Thus, detailed bioinformatic information on the sequence acquisition methods used and their quality control is essential for taxonomic assignments of such viruses [10,11,100].

The ICTV does not require multiple, unique examples of sequences representing a new virus for taxonomic assignment, although characterization of additional members in such new taxa provides further information on genetic diversity and genome completeness. However, when a single sequence represents a species, it is particularly important to ensure that it depicts the virus genome as accurately and completely as possible [100]. The ICTV acknowledges the challenges for taxonomic classification of viruses discovered by metagenomics [11] and is currently working on specific guidelines for the submission of metagenomic sequences to public databases to facilitate taxonomic classification.

Acquisition of metagenomically derived sequence data in large environmental samples provides the best opportunity to fully explore and evaluate the true genetic diversity of viruses. However, the size and genetic complexity of such libraries and the widely used short read Illumina-based sequencing methods may hamper the assembly of complete genome sequences of viruses within samples. Consequently, much of the reported genetic diversity in such studies is based on phylogenetic comparisons of partial genome sequences, often restricted to reads spanning informative genes, such as the polymerases of riboviriad viruses. Such studies are vital for documenting the extent of virus diversity and, indeed, the completeness or otherwise of the current realm and kingdom structure of the virosphere. Partial sequences can also be used to improve the statistical support of large phylogenies that underlie the classification of viruses into taxonomic groups. However, our consensus view is that, without evidence of completeness, genome sequences obtained in such studies cannot be used as the sole basis for the creation of new taxa, meaning that the majority of viral sequences in current metagenomic datasets do not meet the standards for classification at this time. Further progress in long-read sequencing may help to speed up the acquisition of complete viral sequences with greater confidence in their proper assembly and completeness.

## Conclusions

Our description of the four principles of virus taxonomy—which represent the consensus view of the workshop attendees—and the associated review of the evidence we provide gives a roadmap to the ICTV and its constituent expert committees and Study Groups to further develop and expand virus taxonomy. Implementation of the guidelines will also provide consistency and clarity for virus classification to the wider virology community. We acknowledge the vital contribution that expertise in bioinformatics and phylogenetics applied to virus sequence and structure analysis makes to the ever-expanding virus taxonomy. We also recognize the importance of input from virology experts in developing a comprehensive view of the relationships among viruses at all taxonomic ranks.

While there is an established consensus on the need to incorporate viruses characterized in metagenomic studies into virus taxonomy [9], the outcomes of the 2022 workshop presented here effectively describe how this step can be achieved in practice. We have outlined the development and expansion of a taxonomy that was previously primarily based on disease and other phenotypically centered principles. We propose that virus taxonomy can and should now be based formally upon evolutionary relationships among viruses, with phenotypic properties being used where appropriate to inform the placement of lower ranks. This structure should enable the seamless incorporation of viruses characterized from their genomic sequences alone.

## Abbreviations

BSL: biosafety Level
ds: double-stranded
HCV: hepatitis C virus
HIV-1: human immunodeficiency virus type 1
HIV-2: HIV type 2
HTS: high-throughput sequencing
ICTV: International Committee on Taxonomy of Viruses
LIV: louping ill virus
LUCA: last universal cellular ancestor
MCP: major capsid protein
PDB: protein databank
RdRP: RNA-directed RNA polymerase
RT: reverse transcriptase
SARS-CoV: SARS coronavirus
ss: single-stranded
TBEV: tick-borne encephalitis virus
